# Colonization of *Morus alba *L. by the plant-growth-promoting and antagonistic bacterium *Burkholderia cepacia *strain Lu10-1

**DOI:** 10.1186/1471-2180-10-243

**Published:** 2010-09-20

**Authors:** Xianling Ji, Guobing Lu, Yingping Gai, Huijv Gao, Baoyun Lu, Lingrang Kong, Zhimei Mu

**Affiliations:** 1 College of Forestry, Shandong Agricultural University, Tai'an, Shandong, 271018, China; 2 State Key Laboratory of Crop Biology, Shandong Agricultural University, Tai'an, Shandong, 271018, China

## Abstract

**Background:**

Anthracnose, caused by *Colletotrichum dematium*, is a serious threat to the production and quality of mulberry leaves in susceptible varieties. Control of the disease has been a major problem in mulberry cultivation. Some strains of *Burkholderia cepacia *were reported to be useful antagonists of plant pests and could increase the yields of several crop plants. Although *B. cepacia *Lu10-1 is an endophytic bacterium obtained from mulberry leaves, it has not been deployed to control *C. dematium *infection in mulberry nor its colonization patterns in mulberry have been studied using GFP reporter or other reporters. The present study sought to evaluate the antifungal and plant-growth-promoting properties of strain Lu10-1, to clarify its specific localization within a mulberry plant, and to better understand its potential as a biocontrol and growth-promoting agent.

**Results:**

Lu10-1 inhibited conidial germination and mycelial growth of *C. dematium *in vitro; when applied on leaves or to the soil, Lu10-1 also inhibited the development of anthracnose in a greenhouse, but the effectiveness varied with the length of the interval between the strain treatment and inoculation with the pathogen. Strain Lu10-1 could survive in both sterile and non-sterile soils for more than 60 days. The strain produced auxins, contributed to P solubilization and nitrogenase activity, and significantly promoted the growth of mulberry seedlings. The bacteria infected mulberry seedlings through cracks formed at junctions of lateral roots with the main root and in the zone of differentiation and elongation, and the cells were able to multiply and spread, mainly to the intercellular spaces of different tissues. The growth in all the tissues was around 1-5 × 10^5 ^CFU per gram of fresh plant tissue.

**Conclusions:**

*Burkholderia cepacia *strain Lu10-1 is an endophyte that can multiply and spread in mulberry seedlings rapidly and efficiently. The strain is antagonistic to *C. dematium *and acts as an efficient plant-growth-promoting agent on mulberry seedlings and is therefore a promising candidate as a biocontrol and growth-promoting agent.

## Background

Mulberry (*Morus alba *L.), an important feed crop for silkworms, is widely cultivated throughout subtropical and temperate regions in the world. However, the crop is susceptible to a number of diseases throughout the year [1]. These diseases can lead to deterioration of leaf quality, and consumption of infected leaves by silkworm larvae adversely affects their development and cocoon characters [2]. Mulberry anthracnose, caused by *Colletotrichum dematium*, is a commonly observed disease and has become a serious threat to the production and quality of mulberry leaves in susceptible varieties [3] and thus a major problem in mulberry cultivation. As silkworms are reared on mulberry leaves, improper use of agrochemicals to treat the disease could be hazardous to the worms. Therefore, the use of agrochemicals has not gained wide acceptance in mulberry gardens, and the need for alternative techniques that are safe to silkworms is acutely felt. Biological control of plant pathogens using antagonistic bacteria is a promising strategy and has attracted considerable attention in the efforts to reduce the use of agricultural chemicals [4].

Endophytic bacteria are those that colonize plant tissues internally without showing any external symptoms or negative effects on their host [5]. Research has shown the potential of endophytic bacteria as biocontrol and plant-growth-promoting agents [6-8]. The *Burkholderia cepacia *complex (Bcc) is a diverse group of bacteria commonly found in soil, water, and the rhizosphere; on bodies of animal including humans; and in the hospital environment [9]. As endophytic bacteria, members of Bcc have been isolated from a few crops such as sweet corn, cotton, rice, yellow lupine, and sugarcane [10-13], and *B. cepacia *strains have proved useful as antagonists of plant pests and in increasing the yield of several crop plants [14-16].

Strain Lu10-1 of *B. cepacia *(GenBank, EF546394) is an antagonistic endophyte originally isolated from mulberry (*Morus alba *L.) leaves [17]; however, no attempt has been made to use *B. cepacia *for controlling *C. dematium *infection in mulberry nor its colonization patterns have been studied using GFP reporter or other reporters. The objectives of this study were to evaluate the antifungal and plant-growth-promoting properties of Lu10-1, to clarify its specific localization within a mulberry plant, and to better understand its potential as a biocontrol and growth-promoting agent.

## Results

### Antifungal activity of strain Lu10-1 against *C. dematium* in vitro

**Figure 1:**
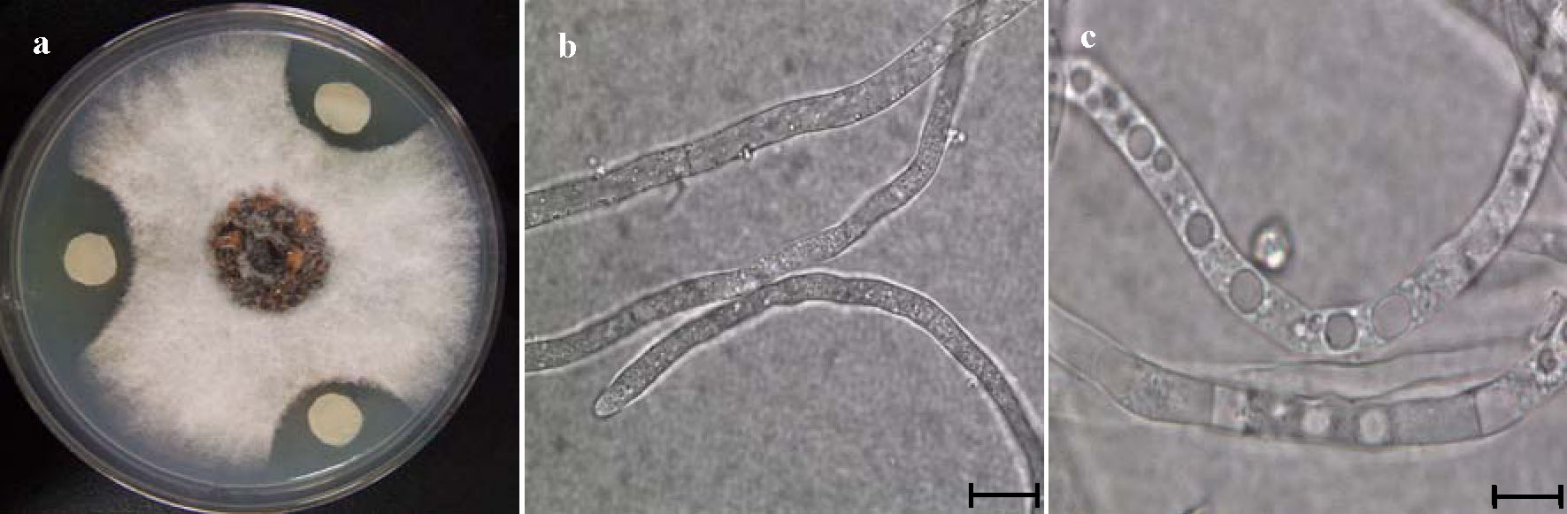
***Burkholdria cepacia* strain Lu10-1 antagonism against *C. dematium in vitro***. a: Interaction between Lu10-1 and *C. dematium *on a PDA plate. b: Microscopic observation of normal *C. dematium *mycelium (Bar = 40 μm). c: Microscopic observation of *C. dematium *mycelium in the zone of interaction with Lu10-1 strains (Bar = 40 μm).

**Figure 2:**
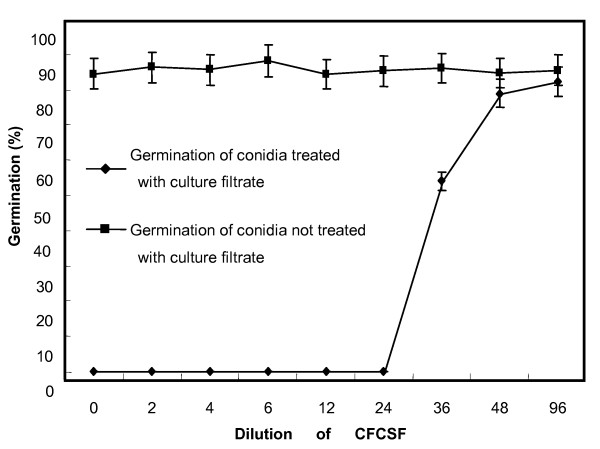
**Germination rates of *C. dematium *conidia in dilutions of CFCSF of strain Lu10-1**. The undiluted filtrate was labelled 0-fold. Sterile liquid LB medium that had not been inoculated with Lu10-1 was placed as control. Each plotted value represents the average of three replicates. Error bars represent SD.

When *C. dematium *and Lu10-1 bacteria were co-cultured on the same PDA plate, a distinct zone of inhibition was observed around the bacterial inoculum (Fig. 1a). Microscopic observation of the hyphae growing close to Lu10-1 colonies showed changes in hyphal morphology such as excessive branching, irregular swelling, curling of hyphal tips, and disruption of apical growth. Mycelium from the co-cultures showed coagulation of cytoplasm, degradation of the mycelium, and large vesicles inside the cell walls (Fig. 1c). Fig. 2 shows the germination rate of conidia suspended in cell-free culture supernatant fluid (CFCSF), undiluted and in a series of dilutions. No conidia could germinate in suspensions containing CFCSF diluted up to 24-fold; at dilutions higher than that, the inhibitory effect decreased, and ceased altogether when the CFCSF was diluted 96-fold.

### Biological control of Lu10-1 against mulberry anthracnose in a greenhouse

**Figure 3:**
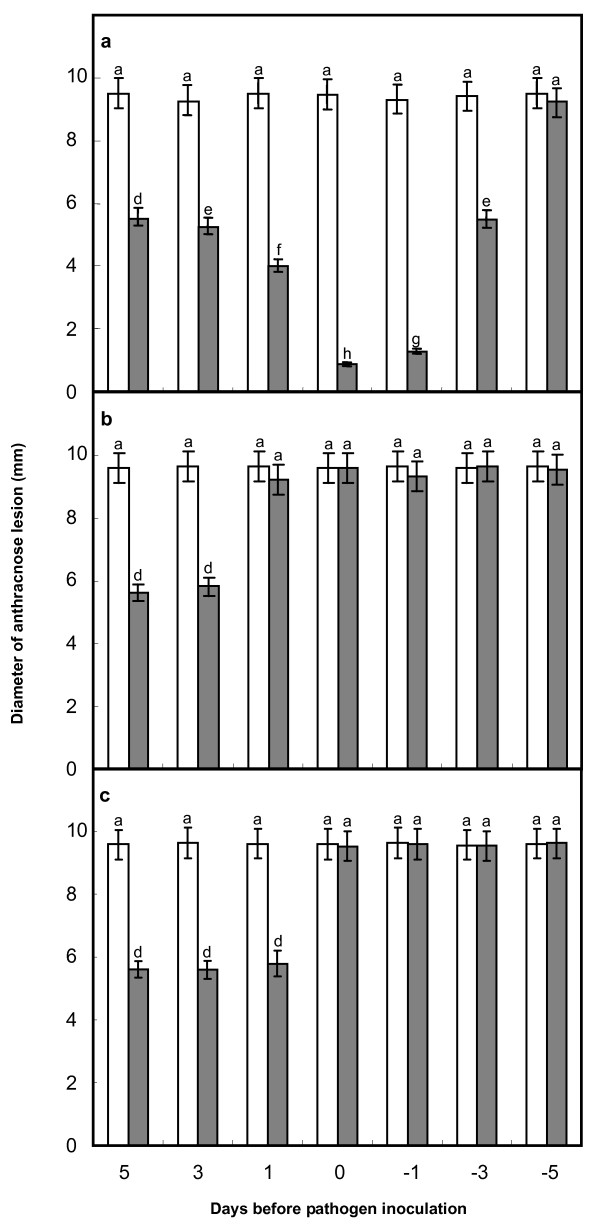
**Efficiency of strain Lu10-1 introduced before or after inoculation with *C. dematium *in controlling mulberry anthracnose in a greenhouse**. (a) Lu10-1 applied to the leaves inoculated with *C. dematium*. (b) Lu10-1 applied to uninoculated leaves. (c) Lu10-1 applied by drenching the soil. Grey columns indicate treatment with Lu10-1 strains and white columns indicate treatment with LB medium (as control). Data are the average of four experiments for three test spots and analyzed using Student's *t*-test (*P *≤ 0.05). Error bars represent SD. The lowercase letters indicate values, with 'a' being the highest, and 'h' the lowest value. The same letters within a column mean that no significant differences exist between the numbers.

To assess the effect of Lu10-1 on the anthracnose on mulberry leaves, the bacteria were applied to inoculated and uninoculated leaves or to the soil at different times before or after inoculation with *C. dematium*. When Lu10-1 was applied to inoculated leaves before or up to 3 days after inoculation, the appearance of anthracnose symptoms was significantly suppressed but not when it was applied 5 days after inoculation (Fig. 3a). It is particularly noteworthy that the symptoms were also suppressed when Lu10-1 was applied to uninoculated leaves or to the soil. In this case too, the degree of suppression varied with the length of the gap between the Lu10-1 treatment and the inoculation (Fig. 3b and 3c), the effective interval being more than 2 days in the case of leaves and one day in the case of soil; intervals longer than these did not result in greater suppression. Thus, it can be seen that strain Lu10-1 proved to be an effective biological control agent against anthracnose of mulberry in greenhouses, and that the strain's effectiveness varied with the length of the interval between the strain treatment and inoculation with the pathogen.

### Survival of rifampicin-streptomycin-tolerant mutants of Lu10-1 in soils

**Figure 4:**
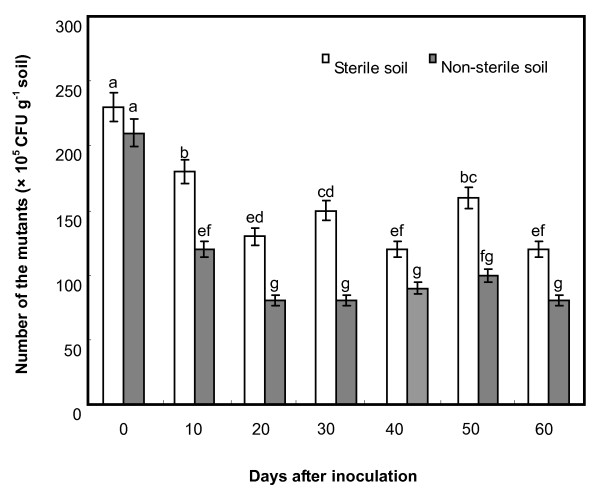
**Survival of Lum10-1 in sterile and non-sterile soil**. The bacterial number was expressed as CFU g^-1 ^dry weight of soils. Data are the average of three experiments and were analyzed using Student's *t*-test (*P *≤ 0.05). Letter 'a' indicates the highest value, and 'g' the lowest value. The same letters within a column mean no significant differences exist between the numbers.

To quantify the survival of rifampicin-streptomycin-tolerant mutants of Lu10-1 (Lum10-1) in soils, Lum10-1 strains were re-isolated from sterile and non-sterile soils at different times after the application (Fig. 4). In sterile soil, over 20 days following the application, the number of bacteria decreased from the initial level of 230 × 10^5 ^CFU g^-1 ^soil to 120 × 10^5 ^CFU g^-1 ^soil. In non-sterile soils, the decrease was both greater and faster. Beyond 20 days, the numbers from both soils remained relatively constant, although significantly higher in the sterile soil. Overall, the Lum10-1 strain could survive in both sterile and non-sterile soils and its population level remained stable for a long time.

### Growth-promoting effects of Lu10-1 on mulberry seedlings

All mulberry seedlings could survive in soils treated with Lu10-1. Seven days after the treatment, the growth of seedlings in the treated soil was not significantly different (*P *≤ 0.05) from that in untreated soil. However, 14 days and 21 days after the treatment, growth was significantly better (*P *≤ 0.05) in the treated soils: the seedlings were taller and the fresh weight of roots and of whole seedlings was greater. No significant differences were found between the seedlings in sterile and non-sterile soils (Table 1). The results indicate significant growth-promoting effect of strain Lu10-1 on mulberry seedlings.

**Table 1:** Plant-growth-promoting effects of Lu10-1 on mulberry seedlings.

Planting soil	Days after inoculation	Height (cm)		Root fresh weight (g/plant)		Seedling fresh weight (g/plant)	
		**Inoculated**	**Control**	**Inoculated**	**Control**	**Inoculated**	**Control**

Sterile soil	7	12.9a^(a)^	12.7a	0.032a	0.032a	0.104a	0.101a
	14	25.4a	18.8b	0.106a	0.071b	0.254a	0.195b
	21	31.5a	22.5b	0.121a	0.082b	0.311a	0.238b

Non-sterile soil	7	13.1a	13.0a	0.040a	0.032a	0.110a	0.109b
	14	24.4a	18.4b	0.107a	0.074b	0.244a	0.195b
	21	31.2a	22.2b	0.120a	0.080b	0.308a	0.236b

(^a^) The same letters within a column mean that no significant differences exist between the numbers; the values are the means of all the seedlings sampled.

### Quantification of endophytic population of Lum10-1 in mulberry seedlings

**Figure 5:**
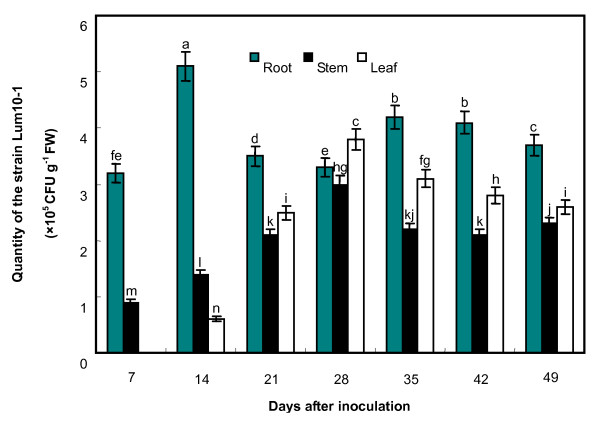
**Population of Lum10-1 in the roots, stems, and leaves of mulberry seedlings**. Student's *t*-test (*P *≤ 0.05) was used to analyse the data. Error bars represent SD. The lowercase letters indicate values, with 'a' being the highest and 'n' the lowest value. The same letters indicate that no significant difference exists between bars. FW indicates fresh weight.

To quantify the endophytic population, Lum10-1 was re-isolated from surface-disinfected roots, stems, and leaves of mulberry seedlings (Fig. 5). The results showed that the bacteria could be re-isolated from surface-sterilized roots and stems on the 7th day after inoculation, implying that the bacteria could successfully establish their presence in roots and stems within 7 days. In the case of leaves, it took 14 days after inoculation, indicating that the bacteria had spread from roots to leaves. Even 49 days after inoculation, the bacteria could be recovered from all parts of the plants, and no damage to the plants was visible. The results of monitoring the growth inside the plants are as follows. The number of bacteria increased initially and fell later, ultimately stabilizing at 1-5 × 10^5 ^CFU per gram of fresh plant tissue. The control seedlings did not yield bacterial colonies when their surface-disinfected roots, leaves, and stems were plated on rifampicin and streptomycin nutrient agar. The above results show that strain Lu10-1 is an endophyte and can spread systemically within mulberry seedling.

### Infection sites of Lu10-1 in mulberry seedlings

**Figure 6:**
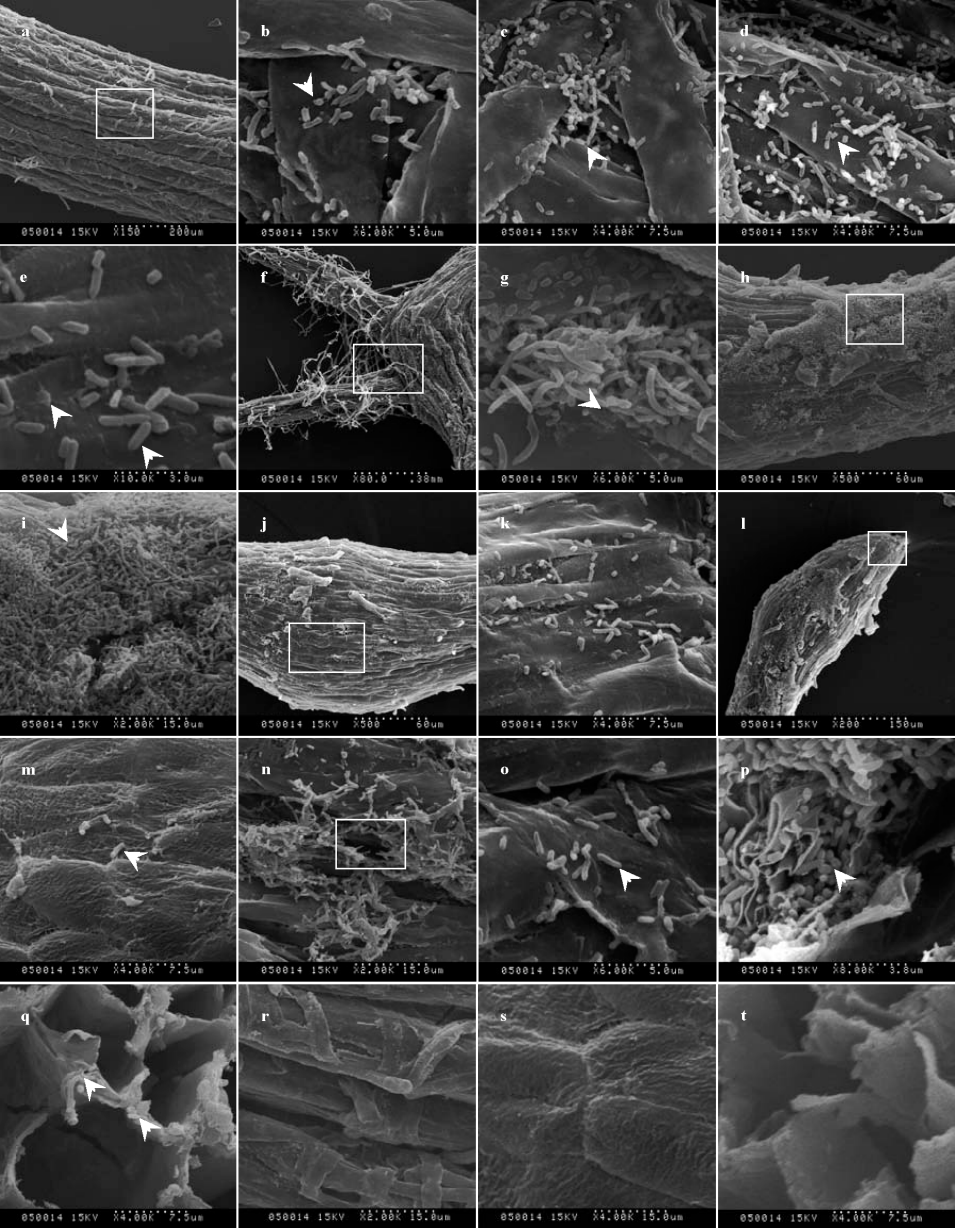
**Scanning electron microscope images of infection sites of Lu10-1 in roots of mulberry seedling**. (a) Colonization of the surface of the root hair zone. (b) Magnified image of the framed region shown in Fig. 5a. (c) Colonization of the sites of root hair emergence. (d) Colonization of the surface of root hair. (e) Lu10-1 cells directly enter the cortex through epidermis in the root hair zone of primary roots. (f) Lu10-1 cells heavily colonize the junctions of primary root with secondary roots. (g) Magnified image of the framed region shown in Fig. 6f. (h) Large-scale colonization of the surface of the zone of elongation. (i) Magnified image of the framed region shown in Fig. 6 h. (j) Colonization of the root meristematic zone. (k) Lu10-1 cells within the depressions formed between epidermal cells as the framed region shown in Fig. 6j. (l) Lu10-1 cells on the surface of the root tip. (m) Magnified image of the framed region shown in Fig. 6l. (n) Lu10-1 cells anchored within the cracks and depressions formed between epidermal cells of primary roots. (o) Magnified image of the framed region shown in Fig. 6n. (p) Numerous cells of Lu10-1 beneath the root epidermis. (q) No bacterial cells were found in the epidermal cells. (r) Zone of root hair in control seedling. (s) Zone of elongation in control seedlings. (t) Optisection of the primary root of a control seedling.

Microscopic observations revealed that the rhizoplane of mulberry seedlings had been colonized by Lu10-1 cells within 24 h of Lu10-1 inoculation of both primary and secondary roots (Fig. 6). The bacteria had colonized the root surfaces in the differentiation, elongation, and root hair zones, as well as the sites from which lateral roots emerge. However, the population density of the bacteria varied with the site: in the root hair zone, the bacterial cells were distributed mainly along the root hair and at the points of their emergence whereas only a few bacteria were observed on the surface of root epidermal cells (Fig. 6a, b, c, and 6d). It is remarkable that some bacteria were found to have entered the cortex directly through the epidermis in this zone (Fig. 6e). We also found that junctions between the primary and the secondary roots had been heavily colonized, indicating that the bacteria enter the roots through the fissures or cracks that are present at the site of emergence of lateral roots and of the radicle (Fig. 6f and 6g). In the elongation zone, surfaces of epidermal cells had been heavily colonized, and the bacteria had formed large cell aggregates (Fig. 6h and 6i), indicating that the elongation zone is another major point of entry. Compared to the elongation zone, the bacteria were sparse in the root meristematic zone, and only single bacterial cells were found within the depressions between adjacent epidermal cells (Fig. 6j and 6k). Similarly, only a few bacterial cells were found on the surface of root tips, a major point of entry into roots for many other microorganisms (Fig. 6l and 6m) [18,19]. Some Lu10-1 bacteria were also observed within the cracks and depressions formed between epidermal cells of primary roots (Fig. 6n and 6o), which is another major entry point for many microorganisms [18,19]. Higher magnifications (Fig. 6p and 6q) revealed that numerous cells of Lu10-1 had colonized the area beneath the root epidermis, but none was found in the epidermal cells. No bacterial cell was observed anywhere on the roots (Fig. 6r, s, and 6t) of the control seedlings. There was no obvious difference between observation taken 24 h and 48 h after inoculation (photographs taken 48 h after inoculation are nor presented).

### Infection process of GFP-tagged Lu10-1 cells in mulberry seedlings

**Figure 7:**
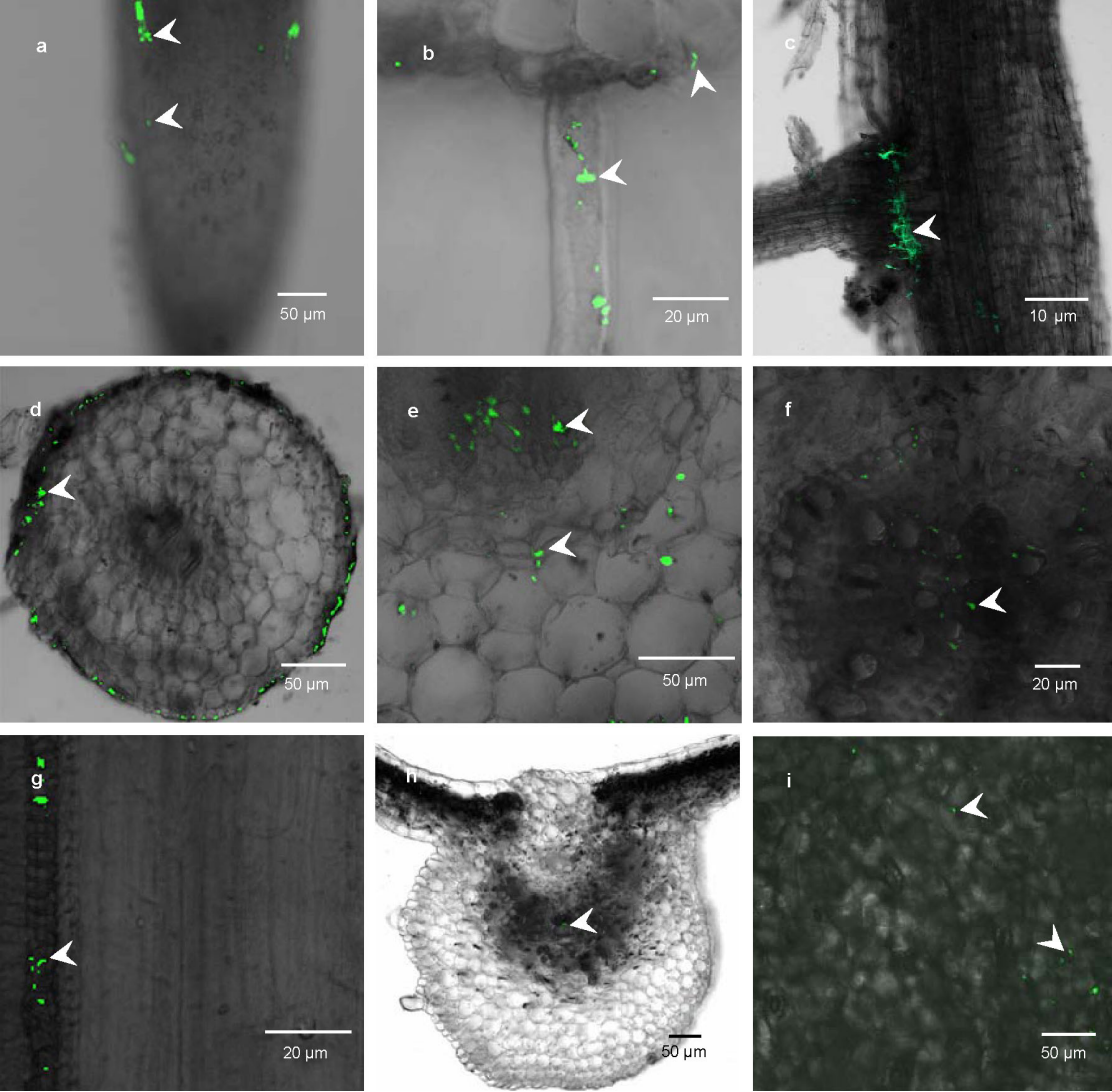
**Confocal laser scanning microscopic images of colonization of mulberry seedlings by Lu10-1 cells tagged with GFP**. (a) Longitudinal section of the primary root showing bacterial cells (arrows) aggregated on root hair and the zone of elongation and sporadic cells in the zone of differentiation and root tip. (b) Transverse section of primary roots showing the bacteria distributed along root hair one day after inoculation. (c) Longitudinal section of the primary root showing the bacteria concentrated at junctions of lateral roots with the primary root one day after inoculation. (d) Transverse section of the primary root showing the labelled bacteria distributed in intercellular spaces of primary root cortical parenchyma 3 days after inoculation. (e) Bacteria had progressed towards inner cortex 5 days after inoculation. (f) Bacteria had colonized the piths of primary roots 7 days after inoculation. (g) Bacteria were found in xylem vessels of stem 11 days after inoculation. (h) Bacteria were found in leaf veins 20 days after inoculation. (i) Bacteria were found in intercellular spaces of leaves 30 days after inoculation.

GFP-labelled Lu10-1 was constructed by transferring an *Escherichia coli - Bacillus cereus *shuttle vector containing the gfp (mut3a) gene into Lu10-1. The labelled Lu10-1 cells emit green fluorescence with excitation and emission wavelengths of 488 and 633 nm, respectively, and could be detected by confocal laser scanning microscopy. After 40 generations in the absence of antibiotic pressure, 65% of the bacteria retained GFP fluorescence, and the expression of gfp did not delay the growth of the transformed strain significantly, which made them suitable for localization studies. The roots, stems, and leaves of mulberry seedlings were optically sectioned at different times after inoculation with GFP-labelled Lu10-1, and examined using a confocal laser scanning microscope. One day after inoculation, the bacterial cells were found to have colonized the surface of the primary roots in the zones of root hair and elongation, and only a few labelled cells were detected in the zones of differentiation and root tip (Fig. 7a). However, labelled Lu10-1 cells were found in large numbers along the root hair (Fig. 7b) and also at the junctions of lateral roots with the main root (Fig. 7c). These results were consistent with the findings observed using the scanning electron microscope (SEM) and confirmed that these bacteria congregate at many entry sites along the length of the root. Three days after inoculation, the bacteria were found in the intercellular spaces of cortical parenchyma of the primary root, and no bacterium was found inside the cells (Fig. 7d). These results are the same as those observed by SEM. The bacteria could be detected in the inner cortex five days after inoculation (Fig. 7e), and could penetrate the pith of the primary root in the next two days (Fig. 7f). At this time, the bacteria were found in the form of cell aggregates in these root tissues, indicating that the process of root infection was complete. Eleven days after inoculation, the bacteria were found in xylem vessels of the stem, indicating that the bacteria had migrated from the root to the stem (Fig. 7g). Twenty days after inoculation, the bacteria were found in leaf veins (Fig. 7h), indicating that the bacterial cells had invaded the leaf. Thirty days after inoculation, the bacteria were observed in the intercellular spaces of leaves, but no bacterium was found inside the cells (Fig. 7i). In contrast, no GFP-labelled Lu10-1 cells were found in the control plants. In summary, our experiments show that the GFP-labelled bacterial cells infect the roots at the zones of differentiation and elongation and through the cracks formed at the junctions between lateral roots and the main root and penetrate the cortex, xylem, and pith. The bacteria can migrate from roots to stems and leaves, and are confined mainly to intercellular spaces.

### Siderophore and indole-3-acetic acid (IAA) production, phosphate solubilization, and nitrogenase activity

Both the qualitative determination of siderophore production and phosphate-solubilizing capacity of Lu10-1 on a solid medium showed positive results, indicating that Lu10-1 can produce siderophores and solubilize phosphates. The rate of nitrogenase activity was 1.16 μmol C_2_H_4 _mg protein^-1 ^h^-1^. Thus, strain Lu10-1 possesses all the plant-growth-promoting characters, namely siderophores, IAA production, P solubilization, and nitrogenase activity.

## Discussion

Our results demonstrate that the strain *B. cepacia *Lu10-1 is an endophyte that can colonize the roots, stems, and leaves of mulberry seedlings rapidly and efficiently following the application of the bacteria by soil drenching. Using GFP-labelled cells *B. cepacia *was found mainly in intercellular spaces of roots and stems, although they were also present within the epidermis, xylem vessels, and cells of the root hair, cortex, and pith. The colonization pattern was similar to that observed for many other endophytes [19-22].

Several mechanisms of disease suppression have been proposed, such as antibiotic metabolites production, siderophore production, and induction of systemic resistance [23]. It was reported that induced systemic resistance (ISR) might be one of the most important operating mechanisms of disease suppression [24,25]. Many investigators have shown that ISR is triggered by bacterial inoculation [26-29]. Our results demonstrate that Lu10-1 is an effective biocontrol agent against anthracnose of mulberry in a greenhouse although the extent of disease suppression varied with the length of the gap between application of the bacterial strain and inoculation with the pathogen (Fig. 3). Although strain Lu10-1 could multiply and spread inside mulberry plants, we could not re-isolate Lu10-1 from the leaves inoculated with *C. dematium *pathogen within 3 days of applying the bacteria either to the soil or uninoculatd leaves. This rules out any physical contact between the bacteria and the pathogen on the leaf surfaces, and yet the plants showed resistance to *C. dematium *at sites distant from the site of application of Lu10-1. We therefore attribute the disease suppression to resistance induced in the mulberry plant, which might be one of the mechanisms underlying biocontrol by Lu10-1. It was reported that bacterial populations must be of certain minimum size before they can induce such resistance [30]. Therefore, some time must elapse between the application of the bacteria and inoculation with *C. dematium *for the bacteria to build up their population to the level necessary for colonizing plant tissues--which is why the extent of disease suppression varied with the length of the interval between the application of Lu10-1 and inoculation with the pathogen. Though the disease was not suppressed when the treatment and the inoculation were simultaneous but the sites of the two interventions were separated in space, it was suppressed significantly when the bacteria were applied to the same site, that is to the inoculated leaves. Furthermore, we found that Lu10-1 produces a metabolite that is released into the medium and inhibits mycelial growth (Fig. 1a) and conidial germination (Fig. 2) in *C. dematium*. Our results show that Lu10-1 can produce bacterial siderophores, which are low-molecular-weight compounds that can inhibit the growth of plant pathogens. These siderophores might also be partly responsible for the biocontrolling properties of Lu10-1. Thus Lu10-1 apparently has multiple mechanisms of antifungal activity that protect mulberry under greenhouse conditions against leaf infection by *C. dematium*. Genetic and biochemical studies will be conducted to determine the exact mechanisms that are essential to the biocontrol potential of strain Lu10-1.

Many endophytic bacteria are known to produce auxins and exhibit P solubilization and nitrogenase activity, all of which enhance root development and improve the uptake of minerals and water [31]. However, bacteria exhibiting all the plant-growth-promoting features simultaneously are rare [32]. Our findings add to this list a novel bacterium, Lu10-1, which has all the plant-growth-promoting characters, namely nitrogenase activity, IAA production, and P solubilization. Plant-growth-promoting effects of Lu10-1 might be due to IAA alone or the combined effects of P solubilization and nitrogenase activity, and future work will elucidate the exact mechanisms.

## Conclusions

Strain Lu10-1 inhibited the development of anthracnose significantly. The strain can survive in both sterile and non-sterile soils for more than 60 days, produces auxins, exhibits P solubilization and nitrogenase activity, and has significant growth-promoting effects on mulberry seedlings. It can also multiply and spread inside mulberry seedlings rapidly and efficiently. Taken together, strain Lu10-1 has great potential as a biocontrol and growth-promoting agent.

## Methods

### Microbial strains

Cultures of *B. cepacia *Lu10-1 and of *C. dematium *were maintained on potato dextrose agar (PDA) [33] plates at 4°C until needed; *C. dematium *was obtained from the Department of Plant Protection of Shandong Agricultural University.

### Evaluation of antifungal activity

Antagonism between Lu10-1 and *C. dematium *was studied by co-culturing the two microorganisms on the same PDA plate. A plug from the edge of an actively growing colony of *C. dematium *was placed at the centre of the PDA plate and a suspension of Lu10-1 at its logarithmic phase growing on Luria-Bertani (LB) medium [34] was added along the periphery. Stock cultures of the bacteria were grown on the LB medium and incubated at 28°C for 1 week and, to prepare the suspension to be used for co-culturing, 100 μL of this stock culture was then added to 100 mL of LB medium and incubated at 37°C while being shaken until the exponential growth phase was reached. The plates with both the organisms were incubated at 25°C for 6-8 d. Plates to which only the LB medium had been added along the periphery served as control. Mycelia in the zone of interaction with Lu10-1 bacteria were removed aseptically from the plates and placed in a drop of sterile water on a glass slide. A coverslip was placed on the film, and observations were made under a microscope (Olympus, Japan).

To evaluate the inhibitory effect of Lu10-1 on the germination of *C. dematium *conidia, the Lu10-1 stock cultures were filtered through a Φ 0.20 μm cellulose acetate membrane (GE Healthcare, USA) filter to obtain the CFCSF. Two-fold series dilution of Lu10-1 CFCSF (10 μL) were placed into two round depressions of a depression glass slide, and 10 μL of sterile liquid LB medium was placed into the two depressions of another glass slide as control. Then, 10 μL of conidial suspension (5 × 10^5 ^conidia mL^-1^) of *C. dematium *was placed into each depression, and the slides were incubated at 25°C and 100% relative humidity (RH) in the dark for 48 h. These preparations were observed under a microscope (Olympus, Japan), and approximately 200 conidia in each depression were examined for germination. A conidium was considered as germinated when the length of its germ tube length was equal to or greater than its diameter. The two depressions on each slide were considered subsamples, and the treatments were replicated three times.

### Evaluation of Lu10-1 as a biocontrol agent

The potential of Lu10-1 to act as a biological agent against mulberry anthracnose in a greenhouse was assessed as described in an earlier paper [35] but with some modifications. Mulberry seedlings used in the experiment were individually planted into 25 cm diameter plastic pots and incubated in a growth chamber at 26°C, 90% RH, and 12 h of light until 5-6 leaves had developed. Two randomly selected leaves from each seedling were used for the test. A filter paper disc (8 mm in diameter) soaked in conidial suspension (2.5 × 10^6 ^conidia mL^-1^) of *C. dematium *was placed on the adaxial surface of the selected leaves. The inoculated leaves were enclosed within polythene bags for 12 h to maintain sufficient humidity. The inoculated leaves were then treated with Lu10-1 applying a suspension of Lu10-1 cells (10^8 ^CFU mL^-1^) with an artist's brush to both surfaces of the leaves. Leaves adjacent to the inoculated leaves were also treated with Lu10-1 similarly, whereas the soil in the pots was treated with Lu10-1 by drenching it with the suspension (12 mL of the suspension per 100 g soil). The gap between inoculation with the fungus and treatment with the bacteria was varied as follows: the leaves or the soil treated (a) 5 d, 3 d, or 1 d before the inoculation; (b) at the same time as the inoculation; and (c) 5 d, 3 d, or 1 d after the inoculation. Seedlings or soils treated only with the LB medium at the same time served as control. The inoculated seedlings were incubated in a greenhouse (approximately 12 h daylight) at 25°C. The seedlings were scored for the disease 10 days after the inoculation based on the diameter of the circular lesions of anthracnose that developed on the inoculated leaves. The test had four replicates and was repeated three times.

### Generation of rifampicin and streptomycin resistant mutants of Lu10-1

Spontaneous chromosomal rifampicin-streptomycin-tolerant mutants of Lu10-1 were generated to quantify the population of Lu10-1 in the soil and in the mulberry plants. First, active cultures of Lu10-1 were plated on LB agar containing 0.1 μg mL^-1 ^of rifampicin and incubated at 25°C until some growth was visible. Single rif^+ ^colonies growing on the plates were selected and purified further by streaking three more times succession on fresh plates of the medium. The purified rif^+ ^mutants were then replated onto nutrient agar containing increasing strengths of rifampicin, and selected step by step the same way until the rif^+ ^mutants that could grow on nutrient agar containing 100 μg mL^-1 ^of rifampicin were obtained. To determine the stability of the mutants, each colony was followed through 10 serial passages on nutrient agar without rifampicin, and rifampicin resistance of each strain confirmed by replating onto nutrient agar amended with rifampicin (100 μg mL^-1^). The rif^+ ^mutants were also compared to the parent strains to ensure that both were morphologically similar as well. These rif^+ ^mutants were then used to select streptomycin-tolerant (100 μg mL^-1^) mutants the same way to obtain the rifampicin and streptomycin resistant mutant, which was designated Lum10-1.

### Quantification of the population surviving in soil

The soil used in this study was collected from the upper 30 cm layer of the mulberry field from which strain Lu10-1 had been isolated. The soil was passed through a 1.5 mm sieve, put into sterilizable polypropylene bags, and autoclaved for 60 min at 120°C four times at 12 h intervals. The autoclaved soil and non-autoclaved soil were brought to about 70% of their maximum water-holding capacity by adding sterile water, drenched with a suspension of Lum10-1 (12 mL of the suspension (10^8 ^CFU mL^-1^) per 100 g soil), packed separately into plastic pots, and maintained in a growth chamber at 26°C, 90% RH, and 12 h of light. At 0, 10, 20, 30, 40, 50 and 60 days after the treatment, 1 g samples of the soils were placed into tubes containing 10 mL of 0.85% (w/v) NaCl solution and agitated in a vortex for 60 s. The suspensions were serially diluted and plated on LB agar containing rifampicin (100 μg mL^-1^) and streptomycin (100 μg mL^-1^). The plates were incubated for 18 h at 37°C, the number of colonies was counted, and the total population was expressed as CFU g^-1 ^of dry weight of the soil. For each treatment, there were four replicates of five samples each. The data were subjected to analysis of variance, and Student's *t*-test was used to estimate the significance of the differences between the means (*P *≤ 0.05).

### Plant-growth-promoting effects of Lu10-1

Healthy mulberry seeds were washed in running tap water for 5 min, surface-disinfected in 20% (w/v) hydrogen peroxide for 3 min and 70% (v/v) ethanol for 90 s, and finally soaked in 10% (w/v) sodium hypochlorite containing 0.01% (v/v) Tween 20 for 3 min. The surface-disinfected seeds were placed on moist filter paper and incubated at 25°C for 5-6 d in Petri dishes. When the roots were about 25 mm long, the seedlings were transplanted into 18 cm diameter plastic pots filled with autoclaved or non-autoclaved soil. Five weeks later, well-rooted and disease-free seedlings were selected for the tests. The seedlings were treated with Lu10-1(10^8 ^CFU mL^-1 ^per 100 g soil) as described above; seedlings treated with sterile distilled water at the same time served as control. All the pots were arranged in a completely randomized design in a growth chamber maitained at 26°C and 14 h of light. The plants were watered as needed. After 7, 14, or 21 days, the shoot length, height, and root weight were recorded. Each experiment was replicated 3 times with 20 pots in each replication.

### Quantification of endophytic population of Lu10-1

Seedlings of mulberry raised as above were incubated in a growth chamber at 26°C, 90% RH, and 12 h of light. When the seedlings were about 10 cm tall, they were treated with Lum10-1 by drenching the soil with a 10^8 ^CFU mL^-1 ^suspension and maintained by watering suitably in a growth chamber as described above. The control seedlings were treated with sterile LB medium. Root, stem, and leaf samples were obtained at different times after the treatment and were surfaced-disinfected as described before [22]. The samples were triturated with a sterile mortar and pestle in potassium phosphate buffer (PB). Serial dilutions of the triturate were made in PB and the cultures grown on nutrient agar containing 100 μg mL^-1 ^of rifampicin and streptomycin. The plates were incubated at 28°C for 48-72 h and colony counts were recorded. For each sampling date, the average of 3 plates of a given dilution was taken for calculating the number of viable cells in 1 mL suspension. For each kind of tissue, there were three replicates with five samples in each replicate. The data were analyzed as described above.

### Infection sites of Lu 10-1 in mulberry seedlings

Mulberry seeds were surface-disinfected and germinated as described above. When no contamination was found on the plates, it was confirmed that the seed surface was sterile. When the roots were about 1 cm long, they were inoculated with Lu10-1 by dipping them in a cell suspension (10^6 ^CFU mL^-1^) for 1 h and then washed with sterile distilled water. Roots of the control seedlings were dipped in sterile distilled water. The treated seedlings were transplanted into 2.5 cm diameter tubes filled with semisolid LB medium and incubated in a plant growth chamber at 25°C under a light regimen comprising 14 h of light alternating with 10 h of darkness. Root samples were obtained at 24 h and 48 h after inoculation. The root samples were fixed in 2.5% glutaraldehyde (v/v) in 0.05 M PB for 2 h, washed in the same buffer, and then fixed in 1% (w/v) osmium tetroxide for 1.5 h. Dehydration was effected with a graded series of ethanol (50%-100%, v/v), and the samples were dried with a critical-point dryer, mounted on stubs, and shadowed with gold (22 nm) for viewing under a SEM (JEM-S570) operating at 20 kV. All images were computer-processed.

### Construction of GFP-labelled Lu10-1 and microscopic observations on colonization in mulberry plant

The plasmid, pGFP4412, containing one copy of constitutively expressed gfp and neomycin- and ampicillin-resistance genes in tandem, was donated by the College of Agronomy and Biotechnology, China Agricultural University, Beijing, China. This plasmid expresses the gfp genes constitutively from the rpsD promoter of *Bacillus subtilis*. The plasmid was introduced into Lu10-1 by electroporation as described in an earlier paper [19]. For transformation, 1 μL of plasmid pGFP4412 was added to 50 μL of competent Lu10-1 cells in a 2 mm electroporation cuvette. The plasmids were electroporated into the cells by using an electroporation system (Bio-Rad) set at 1.6 kV/cm, 25 μF, 200 W, and 416 ms. The transformed cells were immediately transferred to 1 mL of LB medium, incubated for 1 h at 30°C with continuous shaking at 80 rpm, and plated on the selective medium (LB agar containing 7 μg mL^-1 ^neomycin). Transformants, which emitted green fluorescence, were screened with a confocal laser scanning microscope with an excitation wavelength of 488 nm. The stability of the GFP-labelled Lu10-1 was determined as described before [36]. Colonization of mulberry by Lu10-1 was observed with a Bio-Rad MRC1024 confocal laser scanning microscope according to the method described earlier [22]. Images were obtained using Leica confocal software, version 2.477. For each sampling point, six plants were examined. Images were collected from 10-20 sections.

### Estimation of siderophore and IAA production, phosphate solubilization, and nitrogenase activity

Chrome azurole S agar (CAS) was used to assay siderophore production of Lu10-1 as described before [37]. The CAS plates were spot-inoculated with Lu10-1 and incubated at 30°C for 5 days. Development of a yellow-orange halo around the colony was considered as indicative of siderophore production. IAA production was estimated by introducing the bacterial suspension (3 × 10^7 ^CFU mL^-1^) into 10 mL of LB broth containing L-tryptophan (100 μg mL^-1^), incubating the mixture at 30°C for 48 h, and estimating the concentration of IAA in the culture supernatant as described before [38]. P solubilization was tested as described previously [39]. Phosphate-solubilizing activity was considered confirmed when the medium appeared transparent to the eye. Nitrogenase activity was measured by acetylene reduction assay as described before [31] and expressed as micromols of C_2_H_4 _formed per milligram protein per hour.

### Statistics

The data of all experiments were analysed statistically. Confidence intervals are given at 95% limits of confidence. Means were compared with controls by using Student's t-test. Differences were considered significant at the p ≤ 0.05 level.

## Competing interests

The authors declare that they have no competing interests.

## Authors' contributions

XL was responsible for designing the study, collected and prepared the tissues and contributed to write the manuscript. GB carried out antifungal activity analysis of Lu10-1 strain. YP carried out localization analysis of the strain. HJ and BY carried out plant growth-promoting analysis. LR and ZM were responsible for designing the study and contributed to write the manuscript. All authors edited the manuscript and approved the final version.
